# New Regulations of the Secretary of State

**Published:** 1917-02-10

**Authors:** 


### STREET COLLECTIONS.

### New Regulations of the Secretary of State.

By virtue of the powers conferred upon me by Section 5 of the Police, Factories, etc. (Miscellaneous Provisions), Act, 1916, I, being the Police Authority for the Metropolitan Police District, hereby make the following Regulations with respect to the places where and the conditions under which persons may be permitted in any street or public place within the Metropolitan Police District to collect money or sell articles for the benefit of charitable or other purposes:—

(1) No collection of money (other than a collection taken at an open-air meeting) or sale of any article shall be made in any street or public place within the Metropolitan Police District unless the society, committee, or other body of persons responsible for such collection or sale shall have obtained from the Commissioner of Police a permit for such collection or sale. All applications for permits shall be referred to an Advisory Committee appointed by the Commissioner and approved by the Secretary of State.

(2) Application for a permit shall be made in writing in the form contained in the Schedule hereto not later than the first day of the month preceding that month in which it is proposed to hold the collection or sale, and every application shall be made in the name of a committee or other body consisting of not less than three persons, who shall be jointly responsible for the collection or sale.

(3) No collection or sale shall be made except upon the day and between the hours stated in the permit, and no permit of a continuous character will be granted.

(4) The Commissioner may, in granting a permit, limit a collection or sale to such streets or public places or such parts thereof as he may think fit.

(5) No person may assist or take part in any collection or sale without the written authority of the committee or body of persons to whom a permit has been granted. Every person so authorised shall produce such written authority forthwith for inspection by any police officer on demand.

(6) No collection or sale shall be made in any part of the carriage-way of any street, nor shall any collection or sale be made on the footway or in any public place to the obstruction or annoyance of any person in such street or public place.

(7) All persons acting as collectors or vendors shall occupy a stationary position at some place on the footway. Not more than two persons shall act as collectors or vendors at the same place, and no person shall collect money or sell articles within thirty yards of the place where any other person or persons are collecting or selling.

(8) No person under the age of eighteen years shall act or be permitted to act as a collector or vendor.

(9) No collector or vendor shall use a table for the purpose of any collection or sale so as to cause or be likely to cause obstruction. No table used shall exceed 30 inches in length and 20 inches in width.

(10) No collector or vendor shall use a box or other receptacle at the end of a pole intended to reach upper windows or the roofs of conveyances.

(11) No collector or vendor shall be accompanied by any animal.

(12) No collector or vendor shall importune any person to the annoyance of such person.

(13) Every collector or vendor shall carry and present to all contributors or purchasers for the reception of money contributions a box or other receptacle numbered and securely closed and sealed in such a way as to prevent the same being opened or the money extracted without such seal being broken, and into this box or other receptacle all moneys received shall be immediately placed. All such boxes or receptacles shall be numbered consecutively. Every collector or vendor shall deliver his boxes or other receptacles with the seals unbroken to one of the persons responsible for the proper application of the money received.

(14) A collector or vendor shall not carry any collecting box, receptacle, or tray which does not bear displayed prominently thereon the name of the fund for which the collection or sale is being made, nor any box or other receptacle which is not duly numbered.

(15) No payment or reward shall be made to any collector or vendor or other person connected with the promotion or conduct of a collection or sale for or in respect of services rendered in connection therewith.

(16) Within one month after the date of any collection or sale the society, committee, or other body of persons responsible therefor shall forward to the Commissioner for the information of the Advisory Committee a statement in the form contained in the Schedule hereto with vouchers certified by two officials of the society and audited by its auditor or other responsible person not connected with the fund, showing in detail the amount received and the expenses incurred in connection with such collection or sale, and shall, if required by the Commissioner, satisfy him as to the due and proper application of the proceeds of the collection or sale.

Any person who acts in contravention of any of the foregoing Regulations will be liable on summary conviction to a fine not exceeding forty shillings, or in the case of a second or subsequent offence not exceeding five pounds.

The foregoing Regulations do not apply to the selling of articles in any street or public place when the articles are sold in the ordinary course of trade, and for the purpose of earning a livelihood, and no representation is made by or on behalf of the seller that any part of the proceeds of sale will be devoted to any charitable purpose.

I hereby confirm the foregoing Regulations, and direct that the same shall come into operation fourteen days after their publication in the *London Gazette*.*

Geo. Cave,
One of His Majesty's Principal
Whitehall, January 2, 1917. Secretaries of State.

### Schedule.

**Form of Application for Permission to Collect Money or Sell Articles in Streets or Public Places within the Metropolitan Police District for the Benefit of Charitable or Other Purposes.**

1. Name of Society, Committee, or body of persons responsible for the collection or sale.
2. Address.
3. Names and addresses of the applicants for the permit, who will be jointly responsible for the collection or sale.
4. Name of Charity or Fund which is to benefit.
5. Address of the Administrative Centre of the Charity or Fund and name of the Secretary.
6. Objects of the Charity or Fund.
7. Date upon which it is desired to make the collection or sale.
8. Locality within which it is desired to make the collection or sale.
9. The method to be adopted in making the collection or sale.
10. Disposal of the receipts. Are the whole of the receipts to be paid over for the benefit of the Charity or Fund, or will any deduction be made for the expenses or any other purpose? If any deduction is to be made, state for what purpose, and give an estimate of the sum which will be deducted.
11. Signatures of persons making this application.
12. Date of application.

To the Commissioner of Police of the Metropolis.

* These Regulations came into force as from January 19, 1917.

### FORM OF STATEMENT OF RECEIPTS AND EXPENDITURE.

(I.) *In cases where there is no Banking Account.*

Name of Society, Committee, or body responsible for the collection or sale ..........................................

Name of Charity or Fund which is to benefit ..................

Date of collection or sale ..........................................

Statement of Receipts and Expediture.

| Receipts | Amount | Total | Expenditure | Amount | Total |
|---|---|---|---|---|---|
| | £ s. d. | £ s. d. | | £ s. d. | £ s. d. |
| From Street Collections (or sales) as in list of collectors (or vendors) and amounts attached hereto | | | Printing and Stationery ... | | |
| From other sources... ... | | | Postage ... ... | | |
| Donations, as in list attached hereto ... ... | | | Advertising ... | | |
| Bank Interest ... | | | Street Collection Boxes and Carriage ... | | |
| Other items, viz.: | | | Badges or other emblems ... | | |
| | | | Other items (if any):— | | |
| | | | Disposal of Balance (insert particulars) ... | | |

Certified by two officials of the Society ..........................

Names ..........................................................

Addresses ......................................................

Date ..........................................................

Certificate of Auditor, or responsible person not connected with the Society ..........................................

Address ........................................................

Date ..........................................................

### FORM OF STATEMENT OF RECEIPTS AND EXPENDITURE.

(II.) *In cases where there is a Banking Account. All proceeds should be banked immediately and all expenses paid by cheque.*

Name of Society, Committee, or body responsible for the collection or sale ..........................................

Name of Charity or Fund which is to benefit ..................

Date of collection or sale ..........................................

Statement of Receipts and Expenditure.

| Receipts | Amount | Total | Expenditure | Amount | Total |
|---|---|---|---|---|---|
| | £ s. d. | £ s. d. | | £ s. d. | £ s. d. |
| To Street Collections (or sales) as in list of collectors (or vendors) and amounts attached hereto | | | By Bank receipt | | |
| Other sources ... | | | | | |
| Donations, as in list attached hereto ... ... | | | | | |
| Bank Interest ... | | | | | |
| Other items, viz.: | | | | | |

### BANK ACCOUNT.

| Receipts | Amount | Total | Expenditure | Amount | Total |
|---|---|---|---|---|---|
| | £ s. d. | £ s. d | | £ s. d. | £ s. d. |
| To Cash ... ... | | | By Printing and Stationery ... | | |
| | | | Postage ... ... | | |
| | | | Advertising ... | | |
| | | | Street Collection Boxes and Carriage ... | | |
| | | | Badges or other emblems ... | | |
| | | | Other items (if any):— | | |
| | | | Disposal of Balance (insert particulars):— | | |

Certified by two officials of the Society ..........................

Names ..........................................................

Addresses ......................................................

Date ..........................................................

Certificate of Auditor or responsible person not connected with the Society ..........................................

Address ........................................................

Date ..........................................................

